# Point-of-care-ultrasound in in-hospital cardiac arrest

**DOI:** 10.1016/j.resplu.2026.101385

**Published:** 2026-06-10

**Authors:** Bhavya Narala, Kinner Patel, Eyal Menashe, Muhammad Ahsan, Jignesh Patel, Karin Hasegawa, Jie Yang, Sahar Ahmad

**Affiliations:** a Division of Pulmonary, Critical Care and Sleep Medicine, Department of Medicine, Stony Brook University Medical Center, Stony Brook, NY, USA; b Division of Pulmonary and Critical Care, Department of Medicine, University of Alabama Medical Center, Birmingham, AL, USA; c Department of Applied Mathematics and Statistics, Stony Brook University, Stony Brook, NY, USA; d Department of Family, Population & Preventive Medicine, Stony Brook University, Stony Brook, NY, USA

**Keywords:** Ultrasound, Cardiac arrest, Standstill, Survival, CPC, PEA

## Abstract

**Background:**

The purpose of this study was to investigate the relationship between point-of-care-ultrasound (POCUS) findings and survivorship and neurological outcomes at various time points within in-hospital cardiac arrest (IHCA).

**Methods:**

We conducted a single center, prospective, observational study in a tertiary center with data collected between 2014 and 2025. A total of 169 patients were included. Protocolized ultrasound imaging was performed during resuscitation. The primary outcome was the association between different POCUS findings and achievement of ROSC. Secondary outcomes included survival at 1 h, 24 h, 48 h, hospital discharge, and 6 months, and neurological outcomes using cerebral performance category (CPC) scores: 1–2 as good and 3–5 as poor.

**Results:**

Compared to patients with true PEA, pseudo-PEA was associated with improved likelihood of ROSC (OR 3.31, 95% CI 1.52–7.21) and higher survival at 6 months (*p* = 0.003). Cardiac standstill was associated with significantly lower odds of ROSC (OR 0.10, 95% CI 0.05–0.21) and lower survival across all timepoints compared to patients with cardiac activity. Patients without RV stasis demonstrated higher survival proportions at all time points, although this did not reach statistical significance. No statistically significant associations were observed between ultrasound findings and neurological outcomes.

**Conclusion:**

Cardiac standstill and RV stasis were associated with poorer survival in IHCA, while pseudo-PEA was associated with improved survival outcomes, when compared to true PEA. No significant association was observed between POCUS findings and neurological outcomes. Intra-arrest POCUS has implications for survival outcomes, but its role in predicting neurological recovery is an evolving area of investigation, requiring further studies.

## Introduction

The incidence of out-of-hospital cardiac arrest (OHCA) is about 350,000, while that of in-hospital cardiac arrest (IHCA) is about 290,000 annually.^1^, ^2^ While prior studies investigating the role of point-of-care-ultrasound (POCUS) have focused on OHCA, our study is one of the first to investigate the impact of POCUS findings on survivorship and neurological outcomes in the IHCA population. POCUS has long been a diagnostic standard of care tool in the emergency room (ER) setting with particular interest in cardiac arrest.^3^ More and more intensive care unit (ICU) settings have also been implementing this tool, as it is a rapid adjunct tool that may assist in identifying reversible causes of cardiac arrest.^4^ Survivorship to discharge for IHCA is reported to be near to 25%.^1^, ^2^ IHCA survival rates have not changed much over the years despite advances in resources, medicine, and technology, bringing to the forefront the question whether a novel application of an imaging tool, such as POCUS, can confer or predict outcomes. OHCA literature has shown that specific POCUS findings, such as cardiac standstill and pseudo pulseless electrical activity (PEA), can lend to the prediction of varying survival outcomes and related prognosis, at least in the short term.^5^ Although neurological outcomes have been widely studied in the OHCA population, there is unfortunately limited data in IHCA, particularly relating to intra-arrest POCUS findings and longer term neurological recovery. No study has inquired about the aforementioned POCUS findings’ associations to long-term survivorship and neurological outcomes in the IHCA population, nor about the significance of additional POCUS findings such as right ventricular (RV) stasis, until our work.

Cardiac standstill is defined as “complete absence of cardiac motion” at heart walls and valves.^6^ Cardiac standstill in OHCA has been reported to confer a 14% chance of achieving return of spontaneous circulation (ROSC) compared to 51% otherwise.^5^ Congruently, cardiac standstill on POCUS has been shown to confer a positive predictive value of 100% for death in the ER.^7^ RV stasis or “smoke” is defined as echogenic haze in slow repetitive movements (“swirling pattern”) found in low flow conditions.^8^ It is important to distinguish this from microbubble artifact due to active intravenous medications. This artifact typically looks highly echogenic and is rapidly appearing and dissipating, while RV stasis is persistent and slowly swirling. Despite very little being known about this entity, this pattern, when observed in the left heart in the OHCA population, has been suggested to associate with poorer survivorship.^9^ PEA is defined as an absence of palpable pulse with a supraventricular organized electrical activity on the rhythm strip other than ventricular tachyarrhythmias.^10^ In true PEA, there is total electromechanical dissociation, where there is organized electrical activity on the rhythm strip but no visualizable mechanical activity (i.e. cardiac standstill is noted) upon POCUS, while in cases of pseudo-PEA, electrical and mechanical activity (i.e. cardiac activity) are present.^10^, ^11^ These are important to differentiate as there are varying prognostications and may justify future changes within cardiac arrest algorithms.

We sought to determine associations between POCUS findings of cardiac standstill, RV stasis, and pseudo-PEA with survivorship and neurological outcomes at different timepoints in IHCA. Of note, in our study, pseudo-PEA was defined as coordinated cardiac motion on POCUS, while absent cardiac activity or disorganized cardiac motion (i.e. fibrillatory movements) were grouped in with true PEA for analytical purposes. These US findings were selected based on prior literature demonstrating their relevance to cardiac arrest physiology and association with outcomes, particularly in OHCA studies.

## Methods

### Study settings

We conducted a single-center, prospective, protocol-driven, observational study at Stony Brook University Hospital (SBUH) in Long Island, New York. Study data was collected from 2014 through July 2025 and managed using REDCap electronic data capture tools hosted at SBUH. Institutional Review Board approval was obtained (SBUH IRB #302665).

### Subject selection

Adult patients (>18 years) were eligible if they experienced a non-traumatic, pulseless, cardiac arrest event, after admission to the hospital. A protocolized POCUS was performed by trained physicians during resuscitation. Exclusion criteria were incomplete POCUS protocol imaging, duplicate IHCA events for same patients, and resuscitation efforts lasting less than five minutes. Of note, the latter is in accordance with prior IHCA outcome studies.^12^ For patients with duplicate arrest events, only the most recent arrest was included in order to avoid intra-individual clustering and ensure independence of observations. This approach is consistent with observational study methods where a single event per subject is selected to maintain statistical independence. Missing data for all variables were assessed and reported where applicable.

### Study protocol for resuscitation

Ultrasound imaging and interpretation was performed in real time by physicians, specifically fellows or attending from our institution’s critical care services, who were trained by the Echocardiography-boarded division director. Imaging was performed and interpreted during resuscitation using a standardized imaging protocol (Fig. 2) as early as feasible. Typical arrival time, and hence protocol start time, was within 5 min of the call of cardiac arrest. Cardiac activity and other ultrasound findings were assessed using predefined criteria established a priori. Images of the heart were acquired during routine cardiopulmonary resuscitation (CPR) pauses using subxiphoid or apical four-chamber views and were saved for subsequent review. In cases where multiple findings were observed during the arrest, the earliest or predominant finding was used for analysis. Standard advanced cardiac life support (ACLS) processes were not interfered with. During CPR, additional views were observed to rule out reversible causes of cardiac arrest. These included views of bilateral anterior chest wall (rule out pneumothorax), right upper quadrant (rule out hypovolemia/hemorrhagic shock) and anterior neck (confirm endotracheal tube placement). While POCUS views were standardized, order of acquisition was left to the operator. Imaging was saved onto a Health Insurance Portability and Accountability Act (HIPAA) compliant image server, which was accessed by the research team during data collection. Patient related demographics and outcomes data were collected from the electronic health record (EHR). Given the intra-arrest nature of image acquisition, interpretation was not blinded to clinical context or outcomes. No formal inter-rater reliability assessment was performed, and image interpretation was based on the bedside assessment of a trained operator.

**Fig. 1:**
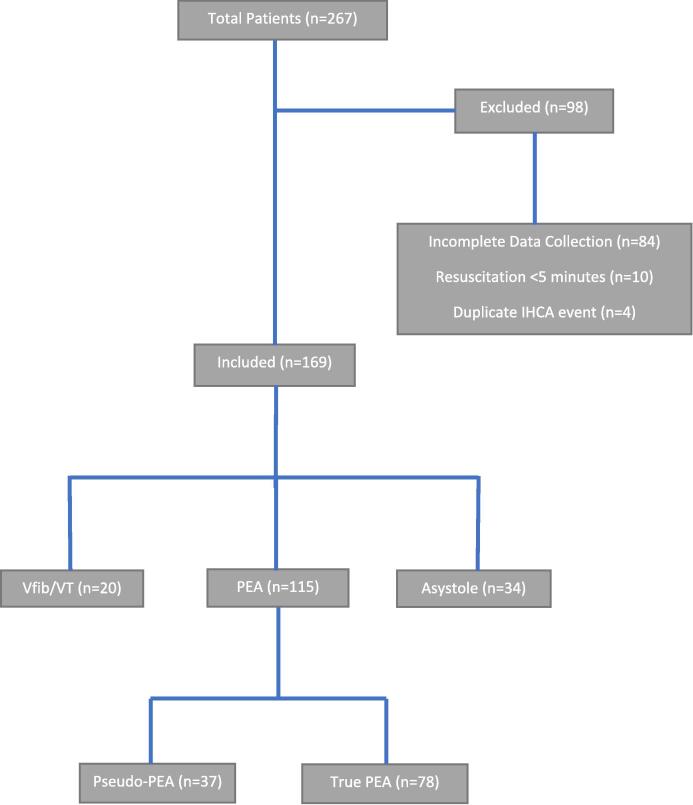
**Flow diagram of participant selection and study enrollment**. Inclusion and exclusion criteria of all patients in our REDCap Database between 2014 and July 2025.

**Fig. 2:**
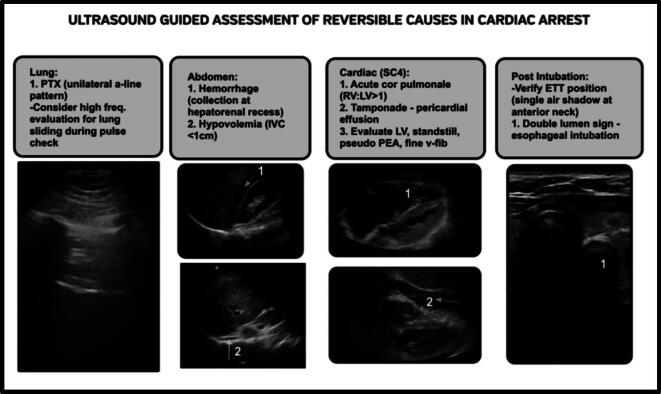
**SBUH POCUS protocol during cardiac arrest: views of bilateral anterior chest wall; right upper quadrant; sub-xiphoid or apical four chamber, anterior neck are included with varying pathology**.

### Study outcomes

The primary outcome was identifying the relationship between different POCUS findings and achievement of ROSC. Secondary outcomes included more timepoints (at 1 h, at 24 h, at 48 h, at hospital discharge, and at 6 months), as well as, neurological status using cerebral performance category (CPC) scores: 1–2 represented good and 3–5 poor neurological outcomes.^13^ POCUS findings were analyzed as independent binary variables and were not mutually exclusive, allowing overlap between categories. CPC scores were obtained using the most updated physician note upon chart review of an individual’s functional status at the various time points (while still in the hospital). Once discharged, telephone calls were performed.

### Statistical analysis

Chi-squared tests with exact *p*-values from Monte-Carlo simulation were utilized to examine the association between the survival/neurological outcomes and the categorical baseline characteristics (gender, initial code rhythm and shockable rhythm). Similar methods were used to examine the association between the US finding variables (pseudo-PEA, cardiac standstill, and RV stasis) and the categorical baseline characteristics, as well as, the US finding variables and the survival/neurological outcomes. Wilcoxon rank sum tests were used to examine the association between the survival/neurological outcomes and the continuous baseline characteristics (age, length of resuscitation and total epinephrine dose). Similar methods were used to examine the association between the US finding variables and the continuous baseline characteristics.

If an ultrasound finding was significantly associated with a particular survival/neurological outcome in the univariate analysis, the association was further examined using a multivariable logistic regression model after adjusting for the baseline characteristics that were significantly associated with the survival/neurological outcome in the univariate analysis. Here, given the limited number of events, we restricted adjusted models to a parsimonious set of clinically relevant variables available in the dataset and associated with resuscitation prognosis. For each survival outcome, the event of interest was ‘Yes (=Survived)’. Similarly, for each neurological outcome, the event of interest was ‘Good’ among those patients who survived. Considering the low event rate of some of the survival and neurological outcomes, forward selection in logistic regression was used, if necessary, where the ultrasound finding variable of the interest was always forced to stay in the model.^14^ In each logistic regression analysis, an OR >1 indicated higher odds of the event outcome, while an OR <1 indicated lower odds of the event outcome. All multivariable regression models are exploratory adjusted analysis and hence all reported *p*-values were not adjusted for multiplicity. *P*-value less than 0.05 was considered statistically significant and analysis was performed using SAS 9.4 (SAS Institute Inc., Cary, NC).

## Results

### Patient characteristics

A total of 267 consecutive IHCA cases were recruited, with 98 excluded due to exclusion criteria (84 due to incomplete POCUS data collection, 10 due to cardiac arrest event lasting less than five minutes, and four due to duplicate events). A total of 169 individuals were included in final analysis (Fig. 1). Of the 169 IHCA events analyzed, 67.5% were male and 32.5% were female (Table 1). The median age was 70 years old. The majority of the population had an initial rhythm of PEA (*n* = 115; 68.1%), followed by asystole (*n* = 34; 20.1%) and Vfib/Vtach (*n* = 20; 11.8%). In the total population, the prevalence of cardiac standstill was 57.4% (*n* = 97) (Table 3) and the prevalence of RV stasis was 33.1% (*n* = 56) (Appendix A, e-Table 10). Of the PEA group (*n* = 115), 37 (32.2%) were pseudo-PEA (Fig. 1). About half of all comers did achieve ROSC (*n* = 85; 50.3%). The median length of resuscitation was 20 min and median dose of epinephrine given was 6 mg. There were no statistical differences noted between survivors and non-survivors in age, gender, and initial rhythm for each time period at ROSC, 1 h, 24 h, 48 h, hospital discharge, and 6 month survival (Appendix A, e-Tables 1–6). Additionally, there were no statistically significant differences noted in age, gender, and initial rhythm between patients with pseudo-PEA and true PEA, (Appendix A, e-Table 7), cardiac standstill and absence of cardiac standstill (Appendix A, e-Table 8), or RV stasis and absence of RV stasis (Appendix A, e-Table 9). Eight (4.7%) individuals had interventions based upon POCUS findings (Table 1), eluding to reversible causes of cardiac arrest, such as pneumothorax indicating a chest tube placement (*n* = 5), pericardial effusion with tamponade physiology indicating a pericardial drain (*n* = 1), and enlarged RV:LV ratio with D-sign seen prompting tissue plasminogen activator (TPA) being given (*n* = 1). One individual was placed on VA ECMO. Of the eight patients who had interventions performed based on POCUS findings, two survived until hospital discharge. Out of the total population group (*n* = 169), 12 (7.1%) individuals survived until the 6 month mark.

**Table 1:** Patient demographics.

**Variable**		**Total (*N* = 169)**
Age		70.00 ± 21.00
Sex	Male	114 (67.46%)
	Female	55 (32.54%)
Initial code rhythm	Asystole	34 (20.12%)
	PEA	115 (68.05%)
	VFib/Vtach	20 (11.83%)
Shockable rhythm	Yes	60 (35.50%)
	No	109 (64.50%)
Length of resuscitation		20.00 ± 17.00
Total epinephrine dose		6.00 ± 5.00
Cardiac standstill	Yes	97 (57.40%)
	No	72 (42.60%)
ROSC achieved	Yes	84 (49.70%)
	No	85 (50.30%)
Interventions*	Yes	8 (4.73)

For categorical variables, percentages across the levels were reported. For continuous variables, median and IQR were reported.

* Interventions were based upon reversible causes of cardiac arrest including but not limited to: chest tube placement, pericardial drain, tissue plasminogen activator administration, ECMO.

**Table 2:** Association between each survival outcome and the presence of pseudo PEA based on univariate models.

**Outcome**		**Total** **(*N* = 115)**	**Pseudo PEA** **(*N* = 37)**	**True PEA** **(*N* = 78)**	***P*-value**
ROSC achieved	Yes	58 (50.43%)	24 (64.86%)	34 (43.59%)	0.04
	No	57 (49.57%)	13 (35.14%)	44 (56.41%)	

1 h survival (within)	Yes	40 (34.78%)	16 (43.24%)	24 (30.77%)	0.21
	No	75 (65.22%)	21 (56.76%)	54 (69.23%)	

24 h survival (at)	Yes	26 (22.61%)	12 (32.43%)	14 (17.95%)	0.10
	No	89 (77.39%)	25 (67.57%)	64 (82.05%)	

48 h survival (at)	Yes	25 (21.74%)	12 (32.43%)	13 (16.67%)	0.09
	No	90 (78.26%)	25 (67.57%)	65 (83.33%)	

Hospital discharge survival (to)	Yes	13 (11.30%)	7 (18.92%)	6 (7.69%)	0.12
	No	102 (88.70%)	30 (81.08%)	72 (92.31%)	

6 month survival (at)*	Yes	7 (6.14%)	6 (16.67%)	1 (1.28%)	0.003
	No	107 (93.86%)	30 (83.33%)	77 (98.72%)	

Column percentages across the level of outcome variables were reported, and *p*-values of Chi-squared test were the exact *p*-values from Monte-Carlo simulation.

* 1 patient was missing in this outcome.

**Table 3:** Association between each survival outcome and the presence of cardiac standstill based on univariate models.

**Variable**		**Total** **(*N* = 169)**	**Cardiac standstill** **(*N* = 97)**	**Absence of cardiac standstill** **(*N* = 72)**	***P*-value**
ROSC achieved	Yes	84 (49.70%)	27 (27.84%)	57 (79.17%)	<0.001
	No	85 (50.30%)	70 (72.16%)	15 (20.83%)	

1 h survival (within)	Yes	63 (37.28%)	19 (19.59%)	44 (61.11%)	<0.001
	No	106 (62.72%)	78 (80.41%)	28 (38.89%)	

24 h survival (at)	Yes	37 (21.89%)	11 (11.34%)	26 (36.11%)	<0.001
	No	132 (78.11%)	86 (88.66%)	46 (63.89%)	

48 h survival (at)	Yes	35 (20.71%)	10 (10.31%)	25 (34.72%)	<0.001
	No	134 (79.29%)	87 (89.69%)	47 (65.28%)	

Hospital discharge survival (to)	Yes	18 (10.65%)	5 (5.15%)	13 (18.06%)	0.010
	No	151 (89.35%)	92 (94.85%)	59 (81.94%)	

6 month survival (at)*	Yes	12 (7.14%)	3 (3.09%)	9 (12.68%)	0.029
	No	156 (92.86%)	94 (96.91%)	62 (87.32%)	

Column percentages across the level of outcome variables were reported, and *p*-values of Chi-squared test were the exact *p*-values from Monte-Carlo simulation.

* 1 patient was missing in this outcome.

### Pseudo PEA vs. true PEA

There was a statistically significant difference between patients with pseudo-PEA and true PEA with respect to achievement of ROSC (*p*-value = 0.04) and 6 month survivorship (*p*-value = 0.003) from univariate analyses, where patients who had true PEA were more likely to not achieve ROSC or survive at 6 month (Table 2). For ROSC achieved, the presence of pseudo PEA remained significant (OR = 3.31, 95% CI: 1.52–7.21, *p*-value = 0.003) after adjusting for length of resuscitation and total epinephrine dose based on a multivariable logistic regression model (Table 4). No further analysis using a multivariable regression model was performed for the 6-month survival outcome due to the small event size (*n* = 7).

**Table 4:** OR with 95% CI for a patient survival outcome based on multivariable logistic regression models.

**Outcome**	**Variable**		**OR (95% CI)**	***P*-value**
ROSC achieved	Pseudo PEA	Yes vs No	3.31 (1.52, 7.21)	0.003
	Length of resuscitation		0.99 (0.96, 1.03)	0.64
	Total Epinephrine dose		0.869 (0.759, 0.995)	0.04

ROSC achieved	Cardiac standstill	Yes vs No	0.10 (0.05, 0.2)	<0.001
	Length of resuscitation		0.98 (0.94, 1.02)	0.28
	Total Epinephrine dose		0.93 (0.80, 1.07)	0.29

1 h survival (within)	Cardiac standstill	Yes vs No	0.17 (0.08, 0.34)	<0.001
	Length of resuscitation		1.00 (0.96, 1.04)	0.89
	Total Epinephrine dose		0.91 (0.79, 1.05)	0.20

24 h survival (at)	Cardiac standstill	Yes vs No	0.27 (0.12, 0.61)	0.002
	Length of resuscitation		0.97 (0.93, 1.02)	0.24
	Total Epinephrine dose		0.87 (0.71, 1.05)	0.14

48 h survival (at)	Cardiac standstill	Yes vs No	0.25 (0.11, 0.58)	0.001
	Length of resuscitation		0.98 (0.93, 1.02)	0.29
	Total Epinephrine dose		0.89 (0.73, 1.07)	0.21

*P*-values were from Type 3 analysis based on a multivariable logistic regression model.

### Cardiac standstill vs. cardiac activity

There was a statistically significant difference between patients with cardiac standstill vs. cardiac activity with respect to achievement of ROSC (*p*-value < 0.001), 1 h (*p*-value < 0.001), 24 h (*p*-value < 0.001), 48 h (*p*-value < 0.001), hospital discharge (*p*-value = 0.010), and 6 month (*p*-value = 0.029) from univariate analyses (Table 3). Patients with cardiac activity were significantly more likely to have survived to each given time point mentioned above as compared to those with cardiac standstill (Table 3). For ROSC, 1 h, 24 h and 48 h time points’ survival, the presence of cardiac standstill remained significant (ROSC achieved: OR = 0.10, 95% CI: 0.05–0.21, *p*-value < 0.001, 1 h survival: OR = 0.17, 95% CI: 0.08–0.34, *p*-value < 0.001, 24 h survival: OR = 0.27, 95% CI: 0.12–0.61, *p*-value = 0.002, 48 h survival: OR = 0.25, 95% CI: 0.11–0.58, *p*-value = 0.001) after adjusting for length of resuscitation and total epinephrine dose (Table 4). For the survival at hospital discharge and 6 month survival, no further analysis using a multivariable logistic regression model was performed due to the small event size (*n* = 18).

### RV stasis vs. absence of RV stasis

Survival proportions were numerically lower among patients with RV stasis, but there was no statistically significant difference between patients with RV stasis and without RV stasis with respect to survivorship (e-Table 10).

### Neurological outcomes

For all neurological outcomes, the number of events (“good”) was small (fewer than 10 in the entire study cohort) at all six timepoints of all three POCUS findings. There was one patient with missing survival outcomes at the 6-month survival time point as they had not reached that specific time period in order for the research team to document the outcome. This patient was excluded from analysis of the corresponding outcomes. None of the neurological outcomes came out as significantly associated with any of the ultrasound findings from the univariate analysis (Appendix A, e-Tables 11–13).

## Discussion

This study investigated POCUS findings as they relate to IHCA survivorship and neurological outcomes. We compared US findings of cardiac standstill, RV stasis, and pseudo-PEA with survivorship and neurological outcomes post arrest at varying time points.

Our study demonstrated a 10.7% survivorship to hospital discharge (Appendix A, e-Table 5), which aligns with ranges observed nationally for IHCA^15^, ^16^ and exceeds the national range for OHCA.^17^ Of those who achieved ROSC (*n* = 84), 67.86% (*n* = 57) were noted to have cardiac activity of some type throughout the resuscitation event (Table 3). On the other hand, of the IHCA non-survivors (*n* = 85), 82.35% (*n* = 70) were noted to have cardiac standstill on POCUS (Table 3). Subjects with any cardiac activity at any time during the resuscitation event had higher likelihood of ROSC compared to the patients who did not show cardiac activity (i.e. cardiac standstill). This pattern persisted when examining survivors vs. non-survivors at time points up until 6 month survival (Table 3). These findings align with that is known from OHCA literature, that cardiac standstill predicts death.^5^, ^6^, ^18^ Of note, cardiac standstill may reflect late-stage arrest physiology, and therefore its association with poorer outcomes may partly reflect underlying disease severity rather than a causal relationship. Study procedures included imaging with POCUS early in the resuscitation and so this principle is not as applicable in our subject population than would be the case in OHCA, where there are delays related to transporting patients to the care center.

Uniquely, we examined the finding of RV stasis as it relates to survivorship. Of those who achieved ROSC (*n* = 84), 73.80% (*n* = 62) were noted to have no evidence of RV stasis throughout the resuscitation event (e-Table 10). On the other hand, of the subjects who did not achieve ROSC (*n* = 85), 40% (*n* = 34) were noted to have RV stasis on POCUS (e-Table 10). While the statistical significance was not achieved in the survival likelihood difference between the two groups at the time point, the prevalence of survival with individuals who had lack of RV stasis was higher than those with RV stasis and this remained up until 6 month time period (e-Table 10). RV stasis may result from inadequate forward flow of blood and/or compromised left ventricular outflow tract (LVOT) flow due to suboptimal chest compression positioning.^19^ Studies have shown that improper hand or device placement during CPR can lead to compression of the LVOT and aortic valve, thus reducing effecting forward flow.^19^ The poorer outcome noted with cardiac standstill and RV stasis are critical findings in the IHCA population. Once verified by larger studies, changes in cardiac arrest management, including standard use of transesophageal echocardiogram (TEE), and/or more efficient resource utilization in select cases are likely to follow, which can also be beneficial from a monetary standpoint within a hospital.

Survivorship was higher in the pseudo-PEA group compared to true-PEA group at the time points of ROSC (*p* = 0.0447) and at 6 months (*p* = 0.0028) (Table 2). This is a paramount finding of our study as earlier OHCA literature has shown that pseudo-PEA may have higher chance of survival up until hospital discharge.^11^, ^20^ Our study of IHCA concludes that such survivorship benefit, while not seen at hospital discharge, is noted at the long term follow up at 6-month mark. There are no OHCA cases delineating long term survivorship of pseudo-PEA for comparison.

Preceding literature has further examined whether high doses of epinephrine should be considered in cases of pseudo-PEA. Gaspari et al. had investigated outcomes in the OHCA population where pseudo-PEA cases were managed with additional continuous adrenergic agents, compared to those receiving standard ACLS agents. It was concluded that there was improved ROSC and survival to hospital admission, but no difference to hospital discharge.^11^ In our study, doses of epinephrine between pseudo-PEA and true PEA groups were statistically similar (Appendix A, e-Table 7), varying only by 1 mg (6 mg vs. 5 mg, respectively) and thus, we are unable to comment on the utility of additional inotropy for pseudo-PEA. Follow up efforts in this area of study would be needed to ascertain the impact of additional epinephrine for pseudo-PEA.

One of the strengths of our study was the systematic measurement of CPC scores at multiple time points, allowing us to assess both survival and functional neurological outcomes. This combination of post-cardiac arrest outcomes has not previously been reported in affiliation to POCUS. Despite observing higher rates of ROSC and survival to discharge in patients with cardiac activity on POCUS, these improvements did not translate into statistically significant differences in neurologically intact survival. Nevertheless, there was a higher proportion of pseudo-PEA (*n* = 4/5; 80%), cardiac activity (*n* = 8/10; 80%), and absence of RV stasis (*n* = 7/10; 70%) in the subgroup with favorable CPC scores at the 6 month time period (Appendix A, e-Tables 11–13).

### Limitations

A limitation noted in this study is power, which could not be controlled due to the observational design. Although the sample size is relatively small, the effects that were seen regarding US findings and survival were adequate to make a conclusion. Adequately powered, large-cohort studies will be necessary to confirm whether the signal toward improved long-term survival (≥6 months) observed in pseudo-PEA represents a true effect, and to rigorously assess the prognostic contribution of POCUS findings to neurological outcomes. It would be advantageous to expand this pilot paper into a multicenter study to assure generalizability across IHCA treating institutions.

Another limitation is the timing of the ultrasound image acquisition during the cardiac arrest. Some images may have been acquired up to five minutes later than others during the arrest (in order to undergo other life save measures i.e. intubation), as our study protocol did not define the timing or order of image acquisition other than as soon as feasible upon arrival. Some likely biases include selectivity (as incomplete POCUS cases were excluded), conclusion of image findings (as these were operator dependent), and timing variability (as mentioned above). Data on patient comorbidities and the underlying etiology of cardiac arrest were not consistently available and were therefore not included, which may limit interpretation of the observed associations. Future studies incorporating these variables would be important to further contextualize our findings.

This study ran several tests on the same dataset, which raises the possibility of obtaining false positive results. Because the analyses were exploratory in nature, the confidence intervals and *p*-values presented have not been adjusted for multiple comparisons. As such, the results should be considered preliminary and will need to be confirmed through future replication studies.

## Conclusion

Cardiac standstill on POCUS during IHCA was associated with poorer survival, consistent with prior OHCA literature. Pseudo-PEA was associated with improved likelihood of ROSC and longer-term survival at 6 months as compared to true PEA. RV stasis was associated with lower survival outcomes. No statistically significant association was observed between POCUS findings and neurological outcomes. In conclusion, intra-arrest POCUS has implications to survival outcomes for IHCA, but its role in predicting neurological recovery is an evolving area of investigation, requiring further validation through larger, multicenter studies.

## CRediT authorship contribution statement

**Bhavya Narala:** Writing – original draft, Resources, Methodology, Investigation, Data curation, Conceptualization. **Kinner Patel:** Writing – review & editing, Investigation, Data curation. **Eyal Menashe:** Writing – review & editing, Investigation, Data curation. **Muhammad Ahsan:** Writing – review & editing, Investigation, Data curation. **Jignesh Patel:** Writing – review & editing, Supervision, Conceptualization. **Karin Hasegawa:** Visualization, Software, Formal analysis, Data curation. **Jie Yang:** Visualization, Software, Formal analysis, Data curation. **Sahar Ahmad:** Writing – review & editing, Supervision, Methodology, Investigation, Data curation, Conceptualization.

## Declaration of competing interest

The authors declare that they have no known competing financial interests or personal relationships that could have appeared to influence the work reported in this paper.

## Appendix A Supplementary material

Supplementary Appendix The supplementary material provides detailed additional statistical analyses and extended results to support and enhance the findings presented in the main text.
