# Supplementary material for: Point-of-care-ultrasound in in-hospital cardiac arrest
Source: Resusc Plus. 2026 Jun 10;30:101385. doi: 10.1016/j.resplu.2026.101385 (PMC13316169; doi:10.1016/j.resplu.2026.101385)
Supplement: Supplementary Appendix — The supplementary material provides detailed additional statistical analyses and extended results to support and enhance the findings presented in the main text. [file mmc1.docx]

**Appendix A. Supplementary Data**

e-Table 1: Patient characteristics by ROSC achieved: yes vs no

| **Covariate** |  | **Total (N=169)** | **Yes (N=84)** | **No (N=85)** | **P-value** |
| --- | --- | --- | --- | --- | --- |
| Age |  | 70.00 ± 21.00 | 69.00 ± 18.00 | 71.00 ± 23.00 | 0.61 |
| Gender | Male | 114 (67.46%) | 58 (50.88%) | 56 (49.12%) | 0.74 |
|  | Female | 55 (32.54%) | 26 (47.27%) | 29 (52.73%) |  |
| Initial Code Rhythm | Asystole | 34 (20.12%) | 17 (50.00%) | 17 (50.00%) | 0.92 |
|  | PEA | 115 (68.05%) | 58 (50.43%) | 57 (49.57%) |  |
|  | VFib/Vtach | 20 (11.83%) | 9 (45.00%) | 11 (55.00%) |  |
| shockable Rhythm | Yes | 60 (35.50%) | 30 (50.00%) | 30 (50.00%) | 1.00 |
|  | No | 109 (64.50%) | 54 (49.54%) | 55 (50.46%) |  |
| Length of resuscitation |  | 20.00 ± 17.00 | 16.00 ± 17.00 | 25.00 ± 16.00 | <.001 |
| Total Epinephrine dose |  | 6.00 ± 5.00 | 4.00 ± 5.00 | 7.00 ± 4.00 | <.001 |
| For categorical variables, row percentages across ROCS achieved “Yes” vs “No” were reported, and p-values of Chi-squared test were the exact p-values from Monte-Carlo simulation. For continuous variables, median and IQR, and p-values based on Wilcoxon rank sum test were reported. | | | | | |

e-Table 2: Patient characteristics by 1 hour survival: yes vs no

| **Covariate** |  | **Total (N=169)** | **Yes (N=63)** | **No (N=106)** | **P-value** |
| --- | --- | --- | --- | --- | --- |
| Age |  | 70.00 ± 21.00 | 69.00 ± 19.00 | 70.50 ± 23.00 | 0.55 |
| Gender | Male | 114 (67.46%) | 43 (37.72%) | 71 (62.28%) | 0.87 |
|  | Female | 55 (32.54%) | 20 (36.36%) | 35 (63.64%) |  |
| Initial Code Rhythm | Asystole | 34 (20.12%) | 16 (47.06%) | 18 (52.94%) | 0.43 |
|  | PEA | 115 (68.05%) | 40 (34.78%) | 75 (65.22%) |  |
|  | VFib/Vtach | 20 (11.83%) | 7 (35.00%) | 13 (65.00%) |  |
| shockable Rhythm | Yes | 60 (35.50%) | 23 (38.33%) | 37 (61.67%) | 0.87 |
|  | No | 109 (64.50%) | 40 (36.70%) | 69 (63.30%) |  |
| Length of resuscitation |  | 20.00 ± 17.00 | 16.00 ± 17.00 | 23.00 ± 16.00 | 0.004 |
| Total Epinephrine dose |  | 6.00 ± 5.00 | 4.00 ± 5.00 | 6.00 ± 5.00 | <.001 |
| For categorical variables, row percentages across 1 hour survival “Yes” vs “No” were reported, and p-values of Chi-squared test were the exact p-values from Monte-Carlo simulation. For continuous variables, median and IQR, and p-values based on Wilcoxon rank sum test were reported. | | | | | |

e-Table 3: Patient characteristics by 24 hour survival: yes vs no

| **Covariate** |  | **Total (N=169)** | **Yes (N=37)** | **No (N=132)** | **P-value** |
| --- | --- | --- | --- | --- | --- |
| Age |  | 70.00 ± 21.00 | 70.00 ± 28.00 | 70.00 ± 21.50 | 0.46 |
| Gender | Male | 114 (67.46%) | 24 (21.05%) | 90 (78.95%) | 0.84 |
|  | Female | 55 (32.54%) | 13 (23.64%) | 42 (76.36%) |  |
| Initial Code Rhythm | Asystole | 34 (20.12%) | 6 (17.65%) | 28 (82.35%) | 0.84 |
|  | PEA | 115 (68.05%) | 26 (22.61%) | 89 (77.39%) |  |
|  | VFib/Vtach | 20 (11.83%) | 5 (25.00%) | 15 (75.00%) |  |
| shockable Rhythm | Yes | 60 (35.50%) | 14 (23.33%) | 46 (76.67%) | 0.84 |
|  | No | 109 (64.50%) | 23 (21.10%) | 86 (78.90%) |  |
| Length of resuscitation |  | 20.00 ± 17.00 | 15.00 ± 11.00 | 23.00 ± 18.50 | <.001 |
| Total Epinephrine dose |  | 6.00 ± 5.00 | 3.00 ± 2.00 | 6.00 ± 5.00 | <.001 |
| For categorical variables, row percentages across 24 hour survival “Yes” vs “No” were reported, and p-values of Chi-squared test were the exact p-values from Monte-Carlo simulation. For continuous variables, median and IQR, and p-values based on Wilcoxon rank sum test were reported. | | | | | |

e-Table 4: Patient characteristics by 48 hour survival: yes vs no

| **Covariate** |  | **Total (N=169)** | **Yes (N=35)** | **No (N=134)** | **P-value** |
| --- | --- | --- | --- | --- | --- |
| Age |  | 70.00 ± 21.00 | 70.00 ± 28.00 | 70.00 ± 22.00 | 0.46 |
| Gender | Male | 114 (67.46%) | 23 (20.18%) | 91 (79.82%) | 0.84 |
|  | Female | 55 (32.54%) | 12 (21.82%) | 43 (78.18%) |  |
| Initial Code Rhythm | Asystole | 34 (20.12%) | 6 (17.65%) | 28 (82.35%) | 0.88 |
|  | PEA | 115 (68.05%) | 25 (21.74%) | 90 (78.26%) |  |
|  | VFib/Vtach | 20 (11.83%) | 4 (20.00%) | 16 (80.00%) |  |
| shockable Rhythm | Yes | 60 (35.50%) | 13 (21.67%) | 47 (78.33%) | 0.85 |
|  | No | 109 (64.50%) | 22 (20.18%) | 87 (79.82%) |  |
| Length of resuscitation |  | 20.00 ± 17.00 | 15.00 ± 12.00 | 23.00 ± 18.00 | <.001 |
| Total Epinephrine dose |  | 6.00 ± 5.00 | 4.00 ± 2.00 | 6.00 ± 5.00 | <.001 |
| For categorical variables, row percentages across 48 hour survival “Yes” vs “No” were reported, and p-values of Chi-squared test were the exact p-values from Monte-Carlo simulation. For continuous variables, median and IQR, and p-values based on Wilcoxon rank sum test were reported. | | | | | |

e-Table 5: Patient characteristics by hospital discharge survival: yes vs no

| **Covariate** |  | **Total (N=169)** | **Yes (N=18)** | **No (N=151)** | **P-value** |
| --- | --- | --- | --- | --- | --- |
| Age |  | 70.00 ± 21.00 | 74.00 ± 27.00 | 69.00 ± 21.00 | 0.74 |
| Gender | Male | 114 (67.46%) | 13 (11.40%) | 101 (88.60%) | 0.79 |
|  | Female | 55 (32.54%) | 5 (9.09%) | 50 (90.91%) |  |
| Initial Code Rhythm | Asystole | 34 (20.12%) | 2 (5.88%) | 32 (94.12%) | 0.54 |
|  | PEA | 115 (68.05%) | 13 (11.30%) | 102 (88.70%) |  |
|  | VFib/Vtach | 20 (11.83%) | 3 (15.00%) | 17 (85.00%) |  |
| shockable Rhythm | Yes | 60 (35.50%) | 8 (13.33%) | 52 (86.67%) | 0.43 |
|  | No | 109 (64.50%) | 10 (9.17%) | 99 (90.83%) |  |
| Length of resuscitation |  | 20.00 ± 17.00 | 15.00 ± 9.00 | 22.00 ± 18.00 | 0.004 |
| Total Epinephrine dose |  | 6.00 ± 5.00 | 2.50 ± 3.00 | 6.00 ± 5.00 | <.001 |
| For categorical variables, row percentages across hospital discharge survival “Yes” vs “No” were reported, and p-values of Chi-squared were the exact p-values from Monte-Carlo simulation. For continuous variables, median and IQR, and p-values based on Wilcoxon rank sum test were reported. | | | | | |

e-Table 6: Patient characteristics by 6 month survival: yes vs no

| **Covariate** |  | **Total (N=168)** | **Yes (N=12)** | **No (N=156)** | **P-value** |
| --- | --- | --- | --- | --- | --- |
| Age |  | 70.00 ± 21.00 | 66.00 ± 27.00 | 70.00 ± 20.50 | 0.27 |
| Gender | Male | 113 (67.26%) | 9 (7.96%) | 104 (92.04%) | 0.75 |
|  | Female | 55 (32.74%) | 3 (5.45%) | 52 (94.55%) |  |
| Initial Code Rhythm | Asystole | 34 (20.24%) | 2 (5.88%) | 32 (94.12%) | 0.32 |
|  | PEA | 114 (67.86%) | 7 (6.14%) | 107 (93.86%) |  |
|  | VFib/Vtach | 20 (11.90%) | 3 (15.00%) | 17 (85.00%) |  |
| shockable Rhythm | Yes | 60 (35.71%) | 7 (11.67%) | 53 (88.33%) | 0.11 |
|  | No | 108 (64.29%) | 5 (4.63%) | 103 (95.37%) |  |
| Length of resuscitation |  | 20.00 ± 17.00 | 16.50 ± 12.50 | 21.00 ± 18.00 | 0.08 |
| Total Epinephrine dose |  | 6.00 ± 5.00 | 2.50 ± 2.00 | 6.00 ± 4.00 | 0.004 |
| For categorical variables, row percentages across 6 month survival “Yes” vs “No” were reported, and p-values of Chi-squared test were the exact p-values from Monte-Carlo simulation. For continuous variables, median and IQR, and p-values based on Wilcoxon rank sum test were reported. | | | | | |

e-Table 7: Baseline Characteristics of Population with initial PEA rhythm

| **Covariate** |  | **Total (N=115)** | **Pseudo PEA (N=37)** | **True PEA (N=78)** | **P-value** |
| --- | --- | --- | --- | --- | --- |
| Age |  | 72.00 ± 20.00 | 70.00 ± 20.00 | 72.50 ± 18.00 | 0.75 |
| Gender | Male | 80 (69.57%) | 25 (31.25%) | 55 (68.75%) | 0.83 |
|  | Female | 35 (30.43%) | 12 (34.29%) | 23 (65.71%) |  |
| Length of resuscitation |  | 20.00 ± 16.00 | 19.00 ± 13.00 | 20.00 ± 17.00 | 0.74 |
| Total Epinephrine dose |  | 6.00 ± 5.00 | 6.00 ± 5.00 | 5.00 ± 4.00 | 0.30 |
| For categorical variables, row percentages across the pseudo PEA (=initial PEA rhythm with pseudo PEA) and true PEA (=initial PEA rhythm with no pseudo PEA) groups were reported, and p-values of Chi-squared test were the exact p-values from Monte-Carlo simulation. For continuous variables, median and IQR, and p-values based on Wilcoxon rank sum test were reported. | | | | | |

e-Table 8: Baseline Characteristics of Population with Cardiac Standstill vs. Cardiac Activity on Ultrasound

| **Covariate** |  | **Total (N=169)** | **Cardiac Standstill (N=97)** | **Absence of Cardiac Standstill (N=72)** | **P-value** |
| --- | --- | --- | --- | --- | --- |
| Age |  | 70.00 ± 21.00 | 69.00 ± 23.00 | 71.50 ± 20.00 | 0.24 |
| Gender | Male | 114 (67.46%) | 63 (55.26%) | 51 (44.74%) | 0.50 |
|  | Female | 55 (32.54%) | 34 (61.82%) | 21 (38.18%) |  |
| Initial Code Rhythm | Asystole | 34 (20.12%) | 21 (61.76%) | 13 (38.24%) | 0.85 |
|  | PEA | 115 (68.05%) | 64 (55.65%) | 51 (44.35%) |  |
|  | VFib/Vtach | 20 (11.83%) | 12 (60.00%) | 8 (40.00%) |  |
| Length of resuscitation |  | 20.00 ± 17.00 | 22.00 ± 15.00 | 16.50 ± 21.50 | 0.06 |
| Total Epinephrine dose |  | 6.00 ± 5.00 | 6.00 ± 4.00 | 4.00 ± 5.50 | 0.005 |
| Length of resuscitation category | 5-8 mins | 25 (14.79%) | 10 (40.00%) | 15 (60.00%) | 0.037 |
|  | 9-15 mins | 37 (21.89%) | 18 (48.65%) | 19 (51.35%) |  |
|  | >15 mins | 107 (63.31%) | 69 (64.49%) | 38 (35.51%) |  |
| For categorical variables, row percentages across the Cardiac standstill and Non-Cardiac standstill group were reported, and p-values of Chi-squared test were the exact p-values from Monte-Carlo simulation. For continuous variables, median and IQR, and p-values based on Wilcoxon rank sum test were reported. | | | | | |

e-Table 9: Baseline Characteristics of Population with Ultrasound Findings of RV Stasis vs No RV Stasis

| **Covariate** |  | **Total (N=169)** | **RV stasis (N=56)** | **No RV stasis (N=113)** | **P-value** |
| --- | --- | --- | --- | --- | --- |
| Age |  | 70.00 ± 21.00 | 69.50 ± 18.50 | 71.00 ± 22.00 | 0.89 |
| Gender | Male | 114 (67.46%) | 38 (33.33%) | 76 (66.67%) | 1.00 |
|  | Female | 55 (32.54%) | 18 (32.73%) | 37 (67.27%) |  |
| Initial Code Rhythm | Asystole | 34 (20.12%) | 12 (35.29%) | 22 (64.71%) | 0.90 |
|  | PEA | 115 (68.05%) | 38 (33.04%) | 77 (66.96%) |  |
|  | VFib/Vtach | 20 (11.83%) | 6 (30.00%) | 14 (70.00%) |  |
| Length of resuscitation |  | 20.00 ± 17.00 | 24.00 ± 19.50 | 19.00 ± 17.00 | 0.004 |
| Total Epinephrine dose |  | 6.00 ± 5.00 | 7.00 ± 5.00 | 4.00 ± 5.00 | 0.001 |
| For categorical variables, row percentages across the RV stasis and No RV stasis group were reported, and p-values of Chi-squared test were the exact p-values from Monte-Carlo simulation. For continuous variables, median and IQR, and p-values based on Wilcoxon rank sum test were reported. | | | | | |

e-Table 10: Association between each survival outcome and the presence of RV stasis based on univariate models

| **Outcome** |  | **Total (N=169)** | **RV stasis (N=56)** | **No RV stasis (N=113)** | **P-value** |
| --- | --- | --- | --- | --- | --- |
| ROSC Achieved | Yes | 84 (49.70%) | 22 (39.29%) | 62 (54.87%) | 0.07 |
|  | No | 85 (50.30%) | 34 (60.71%) | 51 (45.13%) |  |
| 1 hour survival (within) | Yes | 63 (37.28%) | 17 (30.36%) | 46 (40.71%) | 0.23 |
|  | No | 106 (62.72%) | 39 (69.64%) | 67 (59.29%) |  |
| 24 hour survival (at) | Yes | 37 (21.89%) | 8 (14.29%) | 29 (25.66%) | 0.11 |
|  | No | 132 (78.11%) | 48 (85.71%) | 84 (74.34%) |  |
| 48 hour survival (at) | Yes | 35 (20.71%) | 8 (14.29%) | 27 (23.89%) | 0.16 |
|  | No | 134 (79.29%) | 48 (85.71%) | 86 (76.11%) |  |
| Hospital discharge survival (to) | Yes | 18 (10.65%) | 5 (8.93%) | 13 (11.50%) | 0.79 |
|  | No | 151 (89.35%) | 51 (91.07%) | 100 (88.50%) |  |
| 6 month survival (at)* | Yes | 12 (7.14%) | 3 (5.36%) | 9 (8.04%) | 0.75 |
|  | No | 156 (92.86%) | 53 (94.64%) | 103 (91.96%) |  |
| Column percentages across the level of outcome variables were reported, and p-values of Chi-squared test were the exact p-values from Monte-Carlo simulation. *1 patient was missing in this outcome. | | | | | |

e-Table 11: Association between each neurological outcome and the presence of pseudo PEA based on univariate models

| **Outcome** |  | **Total (N=115)** | **Pseudo PEA (N=37)** | **True PEA (N=78)** | **P-value** |
| --- | --- | --- | --- | --- | --- |
| After ROSC | Good | 1 (1.72%) | 0 (0.00%) | 1 (2.94%) | 1.00 |
|  | Poor | 57 (98.28%) | 24 (100.00%) | 33 (97.06%) |  |
| 1 hour survival (within) | Good | 1 (2.50%) | 0 (0.00%) | 1 (4.17%) | 1.00 |
|  | Poor | 39 (97.50%) | 16 (100.00%) | 23 (95.83%) |  |
| 24 hour survival (at) | Poor | 26 (100.00%) | 12 (100.00%) | 14 (100.00%) | . |
| 48 hour survival (at) | Poor | 25 (100.00%) | 12 (100.00%) | 13 (100.00%) | . |
| Hospital discharge survival (to) | Good | 3 (23.08%) | 3 (42.86%) | 0 (0.00%) | 0.19 |
|  | Poor | 10 (76.92%) | 4 (57.14%) | 6 (100.00%) |  |
| 6 month survival (at) | Good | 5 (71.43%) | 4 (66.67%) | 1 (100.00%) | 1.00 |
|  | Poor | 2 (28.57%) | 2 (33.33%) | 0 (0.00%) |  |
| Column percentages across the level of outcome variables were reported, and p-values of Chi-squared test were the exact p-values from Monte-Carlo simulation. | | | | | |

e-Table 12: Association between each neurological outcome and the presence of cardiac standstill based on univariate models

| **Outcome** |  | **Total (N=169)** | **Cardiac Standstill (N=96)** | **Absence of Cardiac Standstill (N=72)** | **P-value** |
| --- | --- | --- | --- | --- | --- |
| After ROSC | Good | 2 (2.38%) | 0 (0.00%) | 2 (3.51%) | 0.56 |
|  | Poor | 82 (97.62%) | 27 (100.00%) | 55 (96.49%) |  |
| 1 hour survival (within) | Good | 3 (4.76%) | 0 (0.00%) | 3 (6.82%) | 0.55 |
|  | Poor | 60 (95.24%) | 19 (100.00%) | 41 (93.18%) |  |
| 24 hour survival (at) | Good | 2 (5.41%) | 0 (0.00%) | 2 (7.69%) | 0.57 |
|  | Poor | 35 (94.59%) | 11 (100.00%) | 24 (92.31%) |  |
| 48 hour survival (at) | Good | 2 (5.71%) | 0 (0.00%) | 2 (8.00%) | 0.58 |
|  | Poor | 33 (94.29%) | 10 (100.00%) | 23 (92.00%) |  |
| Hospital discharge survival (to) | Good | 6 (33.33%) | 1 (20.00%) | 5 (38.46%) | 0.61 |
|  | Poor | 12 (66.67%) | 4 (80.00%) | 8 (61.54%) |  |
| 6 month survival (at) | Good | 10 (83.33%) | 2 (66.67%) | 8 (88.89%) | 1.00 |
|  | Poor | 2 (16.67%) | 1 (33.33%) | 1 (11.11%) |  |
| Column percentages across the level of outcome variables were reported, and p-values of Chi-squared test were the exact p-values from Monte-Carlo simulation. | | | | | |

e-Table 13: Association between each neurological outcome and the presence of RV stasis based on univariate models

| **Outcome** |  | **Total (N=169)** | **RV stasis (N=56)** | **No RV stasis (N=113)** | **P-value** |
| --- | --- | --- | --- | --- | --- |
| After ROSC | Good | 2 (2.38%) | 1 (4.55%) | 1 (1.61%) | 1.00 |
|  | Poor | 82 (97.62%) | 21 (95.45%) | 61 (98.39%) |  |
| 1 hour survival (within) | Good | 3 (4.76%) | 1 (5.88%) | 2 (4.35%) | 1.00 |
|  | Poor | 60 (95.24%) | 16 (94.12%) | 44 (95.65%) |  |
| 24 hour survival (at) | Good | 2 (5.41%) | 0 (0.00%) | 2 (6.90%) | 1.00 |
|  | Poor | 35 (94.59%) | 8 (100.00%) | 27 (93.10%) |  |
| 48 hour survival (at) | Good | 2 (5.71%) | 0 (0.00%) | 2 (7.41%) | 1.00 |
|  | Poor | 33 (94.29%) | 8 (100.00%) | 25 (92.59%) |  |
| Hospital discharge survival (to) | Good | 6 (33.33%) | 3 (60.00%) | 3 (23.08%) | 0.27 |
|  | Poor | 12 (66.67%) | 2 (40.00%) | 10 (76.92%) |  |
| 6 month survival (at) | Good | 10 (83.33%) | 3 (100.00%) | 7 (77.78%) | 1.00 |
|  | Poor | 2 (16.67%) | 0 (0.00%) | 2 (22.22%) |  |
| Column percentages across the level of outcome variables were reported, and p-values of Chi-squared test were the exact p-values from Monte-Carlo simulation. | | | | | |
